# A task-oriented circuit training in multiple sclerosis: a feasibility study

**DOI:** 10.1186/1471-2377-14-124

**Published:** 2014-06-07

**Authors:** Sofia Straudi, Carlotta Martinuzzi, Claudia Pavarelli, Amira Sabbagh Charabati, Maria Grazia Benedetti, Calogero Foti, Michela Bonato, Eleonora Zancato, Nino Basaglia

**Affiliations:** 1 Neuroscience and Rehabilitation Department, Ferrara University Hospital, Ferrara, Italy; 2 Doctoral Program in Advanced Sciences and Technologies in Rehabilitation Medicine and Sports, Tor Vergata University, Rome, Italy; 3 Physical Medicine and Rehabilitation Department, Istituto Ortopedico Rizzoli, Bologna, Italy

**Keywords:** Task oriented, Rehabilitation, Mobility, Multiple sclerosis

## Abstract

**Background:**

The aim of this study was to evaluate the safety, feasibility and preliminary effects of a high-intensity rehabilitative task-oriented circuit training (TOCT) in a sample of multiple sclerosis (MS) subjects on walking competency, mobility, fatigue and health-related quality of life (HRQoL).

**Methods:**

24 MS subjects (EDSS 4.89 ± 0.54, 17 female and 7 male, 52.58 ± 11.21 years, MS duration 15.21 ± 8.68 years) have been enrolled and randomly assigned to 2 treatment groups: (i) experimental group received 10 TOCT sessions over 2 weeks (2 hours/each session) followed by a 3 months home exercise program, whereas control group did not receive any specific rehabilitation intervention. A feasibility patient-reported questionnaire was administered after TOCT. Functional outcome measures were: walking endurance (Six Minute Walk Test), gait speed (10 Meter Walk Test), mobility (Timed Up and Go test) and balance (Dynamic Gait Index). Furthermore, self-reported questionnaire of motor fatigue (Fatigue Severity Scale), walking ability (Multiple Sclerosis Walking Scale – 12) and health-related quality of life (Multiple Sclerosis Impact Scale – 29) were included. Subjects’ assessments were delivered at baseline (T0), after TOCT (T1) and 3 months of home-based exercise program (T2).

**Results:**

After TOCT subjects reported a positive global rating on the received treatment. At 3 months, we found a 58.33% of adherence to the home-exercise program. After TOCT, walking ability and health-related quality of life were improved (p < 0.05) with minor retention after 3 months. The control group showed no significant changes in any variables.

**Conclusions:**

This two weeks high-intensity task-oriented circuit class training followed by a three months home-based exercise program seems feasible and safe in MS people with moderate mobility impairments; moreover it might improve walking abilities.

**Trial registration:**

NCT01464749

## Background

Multiple sclerosis (MS) is a chronic and progressive neurologic disease that commonly affects young adults worldwide and it represents a major cause of disability over time [1,2]. In this population gait and mobility impairments could have a negative impact on personal activities not only restricted to motor domains and participation but also to quality of life [3,4]. There are several studies showing a benefit of physical therapy on walking function, mobility [5-9] and fatigue [10-12]; for this reason recent reviews and international guidelines recommend exercise for MS population [13,14]. Nonetheless, the task-oriented circuit training (TOCT) approach hasn’t been studied enough in people with MS (PwMS) and gait and mobility disorders. TOCT is based on workstations that reproduce physical activities that the subject usually performs during daily living (i.e. walking, climbing stairs, maintain balance) with the aim of promoting motor learning and task retention. In addition, a major characteristic of this rehabilitative intervention is the exercise intensity that, compared to a conventional physiotherapy session, is nearer to the number of repetitions needed to achieve and maintain motor learning of these movements [15]. Moreover, TOCT has been designed for small groups of subjects requiring only one physiotherapist compared with conventional rehabilitation session in which the ratio is 1:1, giving also the advantage of reducing costs of rehabilitation. Previous studies [16,17] have shown that TOCT is a good method to improve locomotor function and mobility in stroke survivors.

The rationale of our study is based on growing evidences [18] that demonstrate how human adult brain is capable of significant adaptations providing that the quantity (duration and frequency) and quality (task-specificity) of interventions are appropriate to facilitate enhanced neural reorganization and upper limb motor recovery in chronic stroke survivors [19]. Also, recent neuroimaging studies highlighted how adaptive brain plasticity [20,21] and motor learning [22] are preserved even in progressive MS subjects and rehabilitative strategies that harness cortical reorganization of motor function might be an option even in this chronically disabled subjects. Task oriented circuit training (TOCT) is an intensive task-specific intervention, for this reason we designed a pilot randomized-controlled trial: (i) to test the safety, feasibility and acceptability of a two weeks high-intensity task oriented circuit training in multiple sclerosis subjects with moderate gait impairments; (ii) to assess its preliminary effects on walking, mobility, fatigue and quality of life; (iii) to evaluate the adherence to a three months home based training program that followed TOCT; (iv) to determine TOCT effect sizes and the sample needed for a future RCT study.

## Methods

This is a single-blind randomized controlled pilot trial. Subjects were recruited at the outpatient clinic of the Physical Medicine and Rehabilitation Department (Ferrara University Hospital). Informed written consent was obtained from eligible subjects. The research study has been reviewed and approved by the Ferrara University Hospital Ethics Committee and registered in ClinicalTrial.gov database (NCT01464749). The inclusion criteria were: males and females, age 18 to 75, diagnosis of MS (primary or secondary progressive, relapsing-remitting), without relapses in the preceding 3 months, mild to moderate gait impairments referred to Expanded Disability Status Scale (EDSS) score between 4 and 5.5. Subjects were able to walk for at least 100 meters with no constant assistance (cane, crutch or brace) required. The exclusion criteria were: other conditions that may affect motor function, impaired cognitive functioning (Mini Mental Status Examination score less than 24). Subjects were randomized to TOCT (experimental group) or usual care (control group) through a block randomization approach. Eligible subjects were randomly assigned into a 1:1 ratio to receive experimental treatment (task-oriented circuit training), or no specific treatment (usual care). The randomization scheme was generated by using the web site Randomization.com (http://www.randomization.com). The experimental group received 10 task-oriented training sessions (Monday-Friday) over 2 weeks; each session lasted 2 hours. Control group did not receive any intervention.

### Task-oriented circuit training

TOCT included six different workstations in which subjects exercised for 5 minutes in each one (3 minutes of exercises and 2 minutes of rest). During each session, subjects underwent 2 laps that took about 60 minutes (6 workstation × 5 minutes × 2 laps), with 10 minutes of rest after each lap. In addition, walking endurance was trained by 30 minutes walking on the treadmill including rests if necessary. For further details on training protocol see Table 1. This was a progressive circuit and subjects while exercising received feedbacks (visual and auditory) by the physiotherapist. Rests were used to discuss about difficulties and to provide further feedbacks. One session included up to 3 patients and lasted 120 minutes, 5 days/week for 2 weeks.

**Table 1:** Task-oriented circuit training description

Patients per group	3
N. of therapists	1 experienced physiotherapist
Intensity (I)	I: 2 weeks, 5 days per week, 120 minutes
Progression (P)	P: increasing the number of repetitions completed in 3 minutes at a workstation and increasing treadmill speed
Exercises	Workstations:
	1: Step (the patient goes up and down a 20 cm step both with left and right foot)
	2: Slalom (the patient kicks a ball walking through a slalom exercise, formed by 4 cones that are 1 m distant from each other)
	3: Tandem exercise (the patient walks in tandem using a line as a guide. If he/she is not able to put one foot in front of the other, it is allowed to do a wider and longer step as long as it is challenging)
	4: Goals (the patient must touch with the tip of the foot a goal that is positioned on a mirror in front of him. If necessary, he can use a lateral support)
	5: Obstacles (the patient must pass 5 obstacles, 3 of which are 5 cm high)
	6: Long step (the patient must walk performing long steps (at least 40-50 cm long) using some signs on the floor as a guide)
	Subjects exercise for 3 minutes at each workstation followed by a 2 minutes break
	7: Treadmill (the patient should walk up to 30 minutes at speed between 0.9 and 2.9 km/h. The speed is self selected by the subject, who can also take a break if she/he needs to)

### Home-based training program

After the supervised 2 weeks, a home- exercise illustrated brochure was given to subjects so that they could independently train for the following 3 months. It included similar exercises that subjects learned during the 2 weeks, gait training (overground or treadmill), stretching and strengthening exercises. We suggested an independent home training 3 times/week (60 minutes/each session). Our principal aim was to retain positive effects on walking competency and function. Subjects were asked to record in a diary the intensity and duration of exercise; they were allowed to call hospital to have further information and feedbacks.

### Usual care (UC)

The control group (UC) did not receive any specific rehabilitation treatment for gait performance and mobility improvement.

During the entire study, both groups were authorized, at will, to exercise in non-rehabilitative contexts.

### Outcome measures

Outcome measures were both functional tests (gait speed, walking endurance, mobility, balance) and self-reported questionnaires (fatigue, health-related quality of life and walking ability). All measures were assessed the week prior to treatment (T0), the week after the end of TOCT (T1) and the 3 months home-based training program (T2).

### 10 Meter Walk Test (10MWT)

It assesses walking speed over 10 meters. It has been tested on different kind of subjects (both with neurological and orthopaedics conditions), including MS [23,24]. The subject walks in total for 14 meters: 2 m for acceleration and 2 m for deceleration, gait speed is calculated for the 10 m distance between them. The test is performed three times and best trial is used for analysis.

### Six Minute Walk Test (SMWT)

Walking endurance was measured with SMWT, a feasible, reproducible and reliable measure in MS subjects [25]. Subjects were instructed to walk up and down a 20m walkway as far as possible in 6 minute.

### Timed Up and Go test (TUG)

Subjects were given verbal instruction to stand up from a chair, walk 3 meters, cross a line marked on the floor, turn around, walk back, and sit down. Subjects performed 3 trials and their best performance was used for analysis [26].

### Dynamic Gait Index (DGI)

It was developed as a clinical tool to assess gait, balance and risk of fall. It doesn’t evaluate only usual steady-state walking, but also walking during more challenging task (i.e. cross obstacles, slalom). Eight functional walking tests are performed by the subject and scored out of three (maximum total score is 24). Scores of 19 or less have been related to an increased risk of falls [27,28].

### Fatigue Severity Scale (FSS)

Fatigue was monitored through a questionnaire, Fatigue Severity Scale (FSS) which evaluates fatigue in MS and other conditions [29]. Essentially, the FSS consists of answering a short questionnaire that requires the subjects to rate their level of fatigue. Subjects were asked to read each statement and circle a number from 1 to 7, depending on how appropriate they felt the statement applied to them over the preceding week.

### Multiple Sclerosis Walking Scale – 12 (MSWS-12)

This questionnaire assesses the impact of MS on walking ability. It is formed by 12 items, asking the patient about his perception on gait speed, running, confidence ascending/discending stairs, balance and fatigue. As in the MSIS 29, the total score is obtained by the sum of the score of each item (0-5) and then transformed into a value from 0 to 100. Again, a higher score represents a major walking disability [30].

### Multiple Sclerosis Impact Scale – 29 (MSIS-29)

This is an health-rated quality of life questionnaire that assesses the impact of MS on physical and psychological functions. It is formed by 29 items on ADL I and II: 20 about physical activity and 9 of psychological status of the person. Each item can be scored with a value from 0 to 5; total score is given by the sum of all the items and then is transformed in a range from 0 to 100. A higher value correspond to a worse perception of subject’s HRQoL [31].

### Feasibility patient-reported questionnaire

At the end of the 2 weeks, subjects who underwent TOCT received a questionnaire formed by twelve questions on their opinions and acceptability referred to the treatment received (for further details see Additional file 1).

### Biostatistical analysis

Descriptive statistic (mean, median, standard deviation, percentiles and confidence interval) was used to describe sample at T0, T1, T2. Baseline characteristics (sex, age, MS type, MS duration, EDSS score) and clinical tests (SMWT, 10 mWT, TUG, DGI, FSS, MSIS-29 and MSWS-12) were compared among groups to assess the quality of randomization. Graphics frequency and Shapiro-Wilk Test were performed to assess variance in order to fulfill the essential assumptions for parametric statistical analysis for continuous variables. To try reducing Type I error, since variables were normally distributed, we choose ANOVA repeated measures for continuous variables (SMWT, 10 mWT and TUG) and Friedman test [32] for ordinal variables (DGI, MSIS-29 and MSWS-12, FSS) to test within-group differences. Post-hoc analysis have been performed when p < 0.05. Differences in mean changes from baseline (T0) after TOCT (T1) and 3 months home-based exercise (T2) were tested with un-paired t-test or Mann Whitney Test among groups. In order to calculate a sample size for a larger RCT, we used d (Cohen) [33]. An intention-to-treat analysis was carried out, i.e. in case of missing data (lost to follow-up) the last available value was considered (last observation carried forward approach). Statistical analysis was performed with STATA software, statistical software v12.1 college station, Texas. Significance level was set at 0.05.

## Results

**Figure 1:**
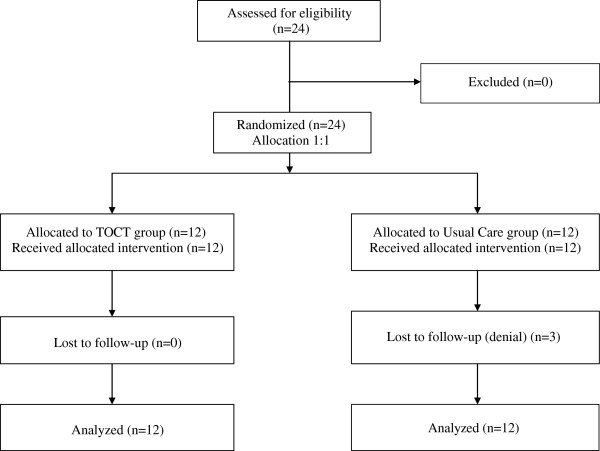
Study flow diagram.

Twenty-four PwMS have been assessed for eligibility in an outpatient rehabilitation clinic (Ferrara University Hospital). After the preliminary visit, all subjects (17 female, 7 male, age 52.58 ± 11.21, 15.21 ± 8.68 years from MS onset) were included in the study. They were randomized into groups: twelve were allocated to TOCT and twelve to UC. Demographic and clinical characteristics are summarized in Table 2. 37.5% of subjects received disease-modifying therapy (DMT) during the entire study (8.33% immunosuppressive treatments, 16.66% interferon beta, 4.16% glatiramer acetate and 8.33% fingolimod); 20.83% of them received therapies for MS symptoms management (i.e. spasticity, depression, pain). We didn’t report significant differences in DMT or symptomatic therapies among groups at baseline or any changes in pharmacological treatments during the study.All subjects completed the 2 weeks training, while we lost 3 subjects at 3 months follow-up in UC group. The clinical study flow-chart has been reported in Figure 1.

**Table 2:** Sample characteristics

		**n**		
	**All subjects**	**TOCT**	**UC**	p value*
Total	24	12	12	
Gender (male/female)	7/17	5/7	2/10	0.18
MS type				0.56
RR	6	4	2	
PP	10	5	5	
SP	8	3	5	
		**Mean (SD) median**		
	**All subjects**	**TOCT**	**UC**	p value**
Age (years)	52.58 (11.21)	49.92 (7.51)	55.25 (13.82)	0.16
	53.50	51.5	58	
MS duration (years)	15.21 (8.68)	12.16 (6.91)	18.25 (9.46)	0.09
	15	10	18	
EDSS score	4.89 (0.54)	4.95 (0.61)	4.83 (0.49)	0.56
	5	5	4.75	

TOCT = task-oriented circuit training. UC = usual care. MS = multiple sclerosis. RR = relapsing remitting. PP = primary progressive. SP = secondary progressive. EDSS = Expanded Disability Status Scale.

*Pearson Chi-square.

**Mann-Whitney test.

### Functional tests (walking endurance, speed, balance, mobility)

A repeated-measures analysis of variance for SMWT, 10MWT and TUG and Friedman test for DGI revealed that TOCT and UC didn’t produce a significant improvement over time. Between-group differences were found in SMWT after TOCT (p < 0.05). Descriptive statistics are shown in Table 3.

**Table 3:** Clinical results

	**TOCT (n=12)**			**UC (n=12)**		
	**Mean (SD)**	**Median (25th-75th Percentile)**	**95% CI**	**Mean (SD)**	**Median (25th-75th Percentile)**	**95% CI**
SMWT (m)						
Pre- TOCT	290.67 (72.92)	283.40 (215.70 – 355.6)	244.33 – 337	285.72 (79.15)	298.40 (230.00 – 329.80)	235.42 – 336.01
Post- TOCT	310.72 (73.73)	316.20 (237.50 – 371.60)	263.87 – 357.57	275.77 (70.65)	293.40 (227.80 – 333)	230.88 – 320.66
3 months follow-up	301.03 (72.58)	308.50 (230.80 – 359.80)	254.92 – 347.15	258.73 (100.80)	274.30 (167.8 – 312.8)	194.69 – 322.78
TUG (s)						
Pre- TOCT	10.64 (2.48)	9.89 (9.06 – 11.85)	9.06 – 12.21	12.10 (4.00)	11.19 (9.24 – 15)	9.56 – 14.63
Post- TOCT	10.05 (1.89)	9.55 (8.56 – 10.93)	8.85 – 11.25	12.23 (3.78)	11.61 (9.27 – 14.07)	9.82 – 14.63
3 months follow-up	10.50 (2.03)	9.56 (9.08 – 11.98)	9.20 – 11.78	12.43 (3.92)	12.25 (9.89 – 14.22)	9.94 – 14.93
10MWT (m/s)						
Pre- TOCT	1.08 (0.23)	1.11 (0.94 – 1.20)	0.94 – 1.23	1.03 (0.30)	0.99 (0.77– 1.19)	0.84 – 1.22
Post- TOCT	1.13 (0.24)	1.10 (1.00 – 1.29)	0.98 – 1.28	1.02 (0.23)	1.03 (0.81 – 1.15)	0.87 – 1.16
3 months follow-up	1.15 (0.19)	1.19 (1.01 – 1.30)	1.02 – 1.27	0.99 (0.30)	0.87 (0.82 – 1.14)	0.80 – 1.19
DGI						
Pre- TOCT	16.83 (3.56)	17 (15.5 – 19.5)	14.57 – 19.10	14.75 (4.92)	14 (12.5 – 18)	11.62 – 17.88
Post - TOCT	17.67 (3.11)	18 (16 – 20)	15.69 – 19.65	14.75 (4.14)	16 (12 – 18)	12.12 – 17.38
3 months follow-up	17.75 (3.93)	18 (14.5 – 10.5)	15.25 – 20.25	13.75 (3.86)	14 (11.5 – 17)	11.29 – 16.21

TOCT = task-oriented circuit training; UC = usual care; SMWT = Six Minute Walk Test; TUG = Timed Up and Go test; 10MWT = 10 Meter Walk Test; DGI = Dynamic Gait Index.

### Patient-reported questionnaires (fatigue, walking ability, health-related quality of life)

MSIS-12 scores were improved in TOCT group (F_(2,22)_ = 3.81, p < 0.05). MSIS-29 physical (F_(2,22)_ = 3.66, p < 0.05) and psychological (F_(2,22)_ = 3.55, p < 0.05) score improved in TOCT group and decreased in UC group (F_(2,22)_ = 3.66, p < 0.05; F_(2,22)_ = 4.28, p < 0.05) over time. Multiple comparisons were significant T0-T1 and T0-T2 in both groups. Between-group differences were found in MSWS-12 and MSIS-29 physical and psychological scores after TOCT (p < 0.01) and in MSIS-29 psychological score even after 3 months of home-based exercise (p < 0.05). Descriptive statistics are shown in Table 4.

**Table 4:** Questionnaire results

	**TOCT (n = 12)**			**UC (n = 12)**		
	**Mean (SD)**	**Median (25th-75th percentile)**	**95% CI**	**Mean (SD)**	**Median (25th-75th Percentile)**	**95% CI**
MSIS-29 Psy (0-100)						
Pre- TOCT	51.66 (20.61)	53.33 (32.22 – 64.44)	38.57 – 64.76	49.44 (20.12)	38.88 (36.11 – 72.22)	36.65 – 62.23
Post- TOCT	41.85 (15.73)	42.22 (30.00 – 46.66)	31.85 – 51.85	53.14 (19.24)	47.77 (40.00 – 72.77)	40.92 – 65.37
3 months follow-up	42.96 (16.20)	43.33 (29.44 – 56.11)	32.66 – 53.25	53.70 (16.43)	50.00 (42.77 – 65.00)	43.26 – 64.14
MSIS-29 Phys (0-100)						
Pre- TOCT	50.83 (15.60)	53.00 (45.50 – 63.50)	40.92 – 60.74	51.58 (18.67)	50.00 (36.25 – 68.50)	39.71 – 63.44
Post- TOCT	44.41 (11.32)	43.00 (39.25 – 50.50)	37.22 – 51.61	53.75 (18.10)	52.00 (38.50 – 70.25)	42.24 – 65.25
3 months follow-up	49.16 (11.00)	48.00 (41.25 – 58.25)	42.17 – 56.15	53.00 (22.28)	49.00 (37.00 – 71.50)	38.83 – 67.16
MSWS-12 (0-100)						
Pre- TOCT	63.06 (14.00)	66.67 (57.50 – 71.67)	54.16 – 71.95	61.94 (18.28)	60 (47.5 – 76.67)	50.33 – 73.56
Post- TOCT	52.36 (14.06)	53.33 (42.5 – 62.5)	43.43 – 61.29	70.83 (17.08)	75 (55 – 82.5)	59.98 – 81.68
3 months follow-up	65.42 (16.04)	63.33 (55 – 76.67)	55.23 – 75.61	71.11 (20.34)	74.17 (55.83 – 88.33)	58.19 – 84.04
FSS						
Pre- TOCT	5.43 (1.23)	5.22 (4.94 – 6.50)	4.65 – 6.22	5.79 (0.98)	6.22 (4.77 –6.55)	5.17 – 6.42
Post- TOCT	5.05 (1.52)	5.44 (4.59 – 6.11)	4.09 – 6.02	6.11 (0.68)	6.11 (5.44 – 6.78)	5.68 – 6.54
3 months follow-up	5.63 (0.78)	5.78 (5.09 – 6.17)	5.13 – 6.13	6.01 (0.91)	6.28 (5.22 – 7)	5.51 – 6.66

TOCT = task-oriented circuit training; UC = usual care; MSIS-29 Psy and Phys = Multiple Sclerosis Impact Scale -29 (psychological and physical functions); MSWS-12 = Multiple Sclerosis Walking Scale – 12; FSS = Fatigue Severity Scale.

### Feasibility patient-reported questionnaire

All subjects (n = 12) completed the questionnaire. On a Visual Analog Scale (VAS) 0-10 they reported a positive global rating on treatment received (9.1), considered the time duration adequate (8.5), the goals were achievable (7.6), the feedbacks provided during sessions were easy to understand (9.9), they enjoyed the training (8.4) and were satisfied by their own results (8.7). At the end of each session they felt physically tired (7), however they didn’t report the need of extra rests (0.25). The majority identified the treadmill (50%) as the exercise where they had best results whereas goals workstation (41.6%) where they had their worst; they considered slalom and tandem exercise workstation (25%) as much more enjoyable, while goals workstation as much more boring (33.33%).

### Home based exercise adherence

At 3 months, 7/12 subjects (58.33%) completed their diary with home-exercise program and returned them to investigators. Considering the SMWT, we didn’t find further gains in subjects who completed the 3 months exercise program (good adherence sub-group) compared with subjects who didn’t report any information on their home exercise training (poor adherence sub-group). Specifically, at three months the good adherence sub-group (n = 7) decreased by -13.28 ± 6.72 m compared to post-TOCT, while the poor adherence sub-group (n = 5) of - 4.64 ± 32.37 m (n.s.).

### Sample size calculation

Our preliminary results allowed us to define a sample size for a following RCT study. Considering walking endurance as primary outcome (SMWT), we found a TOCT effect size (d) of 0.85 in multiple sclerosis subjects (EDSS < 6). This value is based on a walking endurance improvement in TOCT group of 20.05 ± 25.06 m, compared to -9.95 ± 42.84 m in control group. Given equal allocation (1:1) between treatment and control arms, ad using 80% power and alpha of 5%, we would need 46 subjects (23 TOCT + 23 usual care) to complete the study. Conservatively, we expect a 10% rate of drop-out, thus the sample size will be increased by 10% to 51 subjects.

## Discussion

This is a proof-of-concept study with the aim of optimizing components of a novel rehabilitative intervention for PwMS with moderate mobility impairments. We proposed a high-intensity task-specific training which includes several principles of experience-dependent neuroplasticity such as specificity, repetition and intensity [18]; for this reason we hypothesized that a sample of PwMS with moderate mobility impairments might benefit from such intervention. Previous studies on TOCT highlighted positive effects on restoring mobility in stroke survivors including a wide range of exercise doses and intensities [16]. To our knowledge (except for treadmill, body-weight support treadmill and robot-assisted gait training [34]), the only attempts to apply a task-oriented training for mobility impairments in PwMS were done by Mark et al. [35] who proposed a Constraint-Induced Movement Therapy (CIMT) for the lower extremities and Salbach et al. [36] who tested a community exercise program for people with stroke, acquired brain injury and MS. CIMT was administered to a small sample (n = 4) showing positive effects on real-world lower extremity use up to four years. Compared to our TOCT, CIMT protocol was much more intense (3.5 h/d for 3 wk), trained 15 different tasks (compared to 7) and a “transfer package” to deliver gains into the real world was administered [35]. The community exercise program was delivered to 2 MS subjects twice/week for 12 weeks with no effects on walking endurance [36]. Our two weeks high-intensity task oriented circuit training was feasible and safe in a small group of PwMS with moderate mobility impairments. We didn’t report any injuries as a consequence of falls during TOCT sessions or marked fatigability which required the interruption of the exercise; all twelve subjects concluded the 10 sessions with no drop-outs. Furthermore, looking at the feasibility patient-reported questionnaire results, we might conclude that this is a suitable intervention in this population. To test TOCT effects on walking function we did both short-distance (10MWT) than long-distance (SMWT) walking tests in addition to a patient-reported measure of walking ability (MSWS-12). Long-distance walking test and patient-reported walking ability questionnaires were considered as the best measures to detect changes after rehabilitation in MS subjects [37]. Furthermore, long-distance walking tests, such as SMWT better reflect walking performance and functional capacity in MS population [38]. A good predictor for independency and mobility in PwMS is walking endurance measured by SMWT: it has been shown that subjects have 1% increased probability of being independent for every 1 m longer they walk during SMWT [23]. In our sample we didn’t find significant effects on gait speed measured with 10MWT probably because short-distance more than long-distance walking test are due to variability. In fact, a recent study reported a within-day variability of >20% for the 10MWT when baseline gait velocity was <1.2 m/s [39]. Another reason would be that we didn’t use the 25 foot walking test, which seems to be more reliable than 10MWT in detecting changes in PwMS [40]. Our findings in SMWT gains were close to the minimally important change from a patient prospective stated as 22 m [37]. In the same way MSWS-12 score improved by 11 points, showing beneficial effects on walking ability; this patient–reported outcome measure investigates limitation on walking abilities due to MS [30] and seems to be reliable in detecting changes after rehabilitation with a minimally important change of 10.39 points [37]. Considering how health-related quality of life is strongly reduced in PwMS [4], we found positive effects of TOCT on both physical and psychological functions in MSIS-29; however we have to consider how these results are within the standard error of the measurement which is defined as 5.3-9.5 points for physical domain and 7.6-13 points for psychological domain [41,42]. Examining fatigue which is a common MS symptom with a negative impact on mobility, activities and quality of life, in our sample we didn’t find any detrimental effects of the task-oriented circuit training (2h/d over 2 weeks). Accordingly with previous studies [10-12] we highlighted a slight fatigue reduction after TOCT. Nevertheless, these preliminary results should be taken cautiously, considering that this is a pilot study with the aim to test the safety and feasibility of a task-oriented circuit training in PwMS.

In our sample we found a lower adherence to home-exercise program (58%) compared to other studies in MS subjects [43-45]. We can assume that giving a diary with a home-based exercise program wasn’t sufficient to motivate subjects to continue with task-oriented exercises at home. We can also speculate that task-oriented training is less suitable at home compared to other intervention tested in the aforementioned studies (mostly resistance exercises). Moreover, depression or cognitive symptoms, that are common in MS, might have reduced subjects’ compliance to rehabilitation, as clearly highlighted for MS medications [46]. However, we didn’t find any differences in walking endurance deterioration after TOCT among good- and poor-adherence subgroups.

Finally, as long walking tests are more appropriate in detecting clinically meaningful improvement after physical rehabilitation in MS population [37,38], we calculated a sample size for a future RCT study based on the SMWT gains (51 subjects).

### Limits and future directions

Our pilot study has several limitations. Firstly, our small and heterogeneous sample didn’t allow us to draw definite inferences from this analysis; moreover the inability to blind subjects’ on the treatments received (TOCT or UC) might have reduced the internal validity of the study; secondly, we didn’t use neither a valid method to address home-exercise adherence, nor psychiatric and neuropsychological evaluation to identify possible factors related to discontinued therapies; finally, we didn’t have real-world measures (i.e. accelerometer or step counters) to test transferability in everyday life.

Future directions: (i) to conduct a large RCT trial to confirm this preliminary results with a longer follow-up; (ii) to better explain MS mechanisms of functional recovery it would be necessary to add neuroimaging parameters (i.e. functional near-infrared spectroscopy); (iii) to quantify the dose of exercise delivered; (iv) to add real-world based measures (i.e. steps counters and specific patient-reported questionnaires); (v) to test TOCT in a wide range of mobility impairments (EDSS ≥ 6); (vi) to increase home-exercise program adherence with different methods (i.e. customized pamphlets, phone calls, physiotherapy visits).

## Conclusions

In this pilot study, we assessed the safety, feasibility and preliminary effects on selected variables (gait speed, gait endurance, mobility, balance and health-related quality of life) of a task-oriented circuit training in a sample of multiple sclerosis subjects. Our main findings revealed that a 2 weeks task-oriented circuit training followed by a three months home exercise program is safe and well tolerated in multiple sclerosis subjects with moderate gait and mobility impairments. TOCT may allow a more intensive (more repetitions) and specific (task oriented) exercise in an enriched rehabilitative setting. No detrimental effects on subjective fatigue have been observed in this sample or major adverse effects (i.e. fall-related injury). Even though some positive effects on walking ability has been underlined, this trial cannot draw definitive conclusions on the effects of such intervention.

## Abbreviations

TOCT: task-oriented circuit training; MS: multiple sclerosis; HRQoL: Health-related quality of life; EDSS: Expanded Disability Status Scale; PwMS: People with multiple sclerosis; UC: Usual care; 10MWT: 10 Meter Walk Test; SMWT: Six Minute Walk Test; TUG: Timed Up and Go Test; DGI: Dynamic Gait Index; FSS: Fatigue Severity Scale; MSWS-12: Multiple Sclerosis Walking Scale – 12; MSIS-29: Multiple Sclerosis Impact Scale; DMT: Disease-modifying therapy; VAS: Visual Analog Scale; CIMT: Constraint – Induced Movement Therapy.

## Competing interests

The authors declare that they have no competing interests.

## Authors’ contributions

SS, MB, EZ, ASC and NB participated in the design of the study (including TOCT protocol); EZ, ASC, MB and CM carried out clinical tests; SS, CM, ASC, CP, MGB, NB and CF drafted the manuscript; SS and CP performed the statistical analysis. All authors read and approved the final manuscript.

## Pre-publication history

The pre-publication history for this paper can be accessed here:

http://www.biomedcentral.com/1471-2377/14/124/prepub

## Supplementary Material

Additional file 1 Feasibility patient-reported questionnaire. Click here for file
