# Supplementary material for: A task-oriented circuit training in multiple sclerosis: a feasibility study
Source: BMC Neurol. 2014 Jun 7;14:124. doi: 10.1186/1471-2377-14-124 (PMC4059088; doi:10.1186/1471-2377-14-124)
Supplement: Additional file 1 — Feasibility patient-reported questionnaire. [file 1471-2377-14-124-S1.doc]

**Feasibility patient-reported questionnaire**

Dear Sir/Madame, we are collecting opinions about the use of a task-oriented circuit training for walking improvement. You should read the following questions and give your judgment marking the line below each question.

Did you enjoy this kind of training?

**Minimum (0) Maximum (10)**

If not, why? (Report any condition of discomfort)

Was the time exercise adequate?

**Minimum (0) Maximum (10)**

Were the goals of each exercise easy to achieve?

**Minimum (0) Maximum (10)**

Were the instructions given by the therapist easy to understand?

**Minimum (0) Maximum (10)**

How many breaks did you take in addition to those that were given?

In which exercise did you obtain the best results?

In which one did you have the worst results?

Which was the most enjoyable exercise?

Which one was the most boring?

Do you feel physically tired?

**Minimum (0) Maximum (10)**

Did you have fun?

**Minimum (0) Maximum (10)**

Are you satisfied about your performance?

**Minimum (0) Maximum (10)**
